# The development and pilot testing of a rapid assessment tool to improve local public health system capacity in Australia

**DOI:** 10.1186/1471-2458-9-413

**Published:** 2009-11-15

**Authors:** Prue Bagley, Vivian Lin

**Affiliations:** 1 School of Public Health, La Trobe University, Melbourne, Australia

## Abstract

**Background:**

To operate effectively the public health system requires infrastructure and the capacity to act. Public health's ability to attract funding for infrastructure and capacity development would be enhanced if it was able to demonstrate what level of capacity was required to ensure a high performing system. Australia's public health activities are undertaken within a complex organizational framework that involves three levels of government and a diverse range of other organizations. The question of appropriate levels of infrastructure and capacity is critical at each level. Comparatively little is known about infrastructure and capacity at the local level.

**Methods:**

In-depth interviews were conducted with senior managers in two Australian states with different frameworks for health administration. They were asked to reflect on the critical components of infrastructure and capacity required at the local level. The interviews were analyzed to identify the major themes. Workshops with public health experts explored this data further. The information generated was used to develop a tool, designed to be used by groups of organizations within discrete geographical locations to assess local public health capacity.

**Results:**

Local actors in these two different systems pointed to similar areas for inclusion for the development of an instrument to map public health capacity at the local level. The tool asks respondents to consider resources, programs and the cultural environment within their organization. It also asks about the policy environment - recognizing that the broader environment within which organizations operate impacts on their capacity to act. Pilot testing of the tool pointed to some of the challenges involved in such an exercise, particularly if the tool were to be adopted as policy.

**Conclusion:**

This research indicates that it is possible to develop a tool for the systematic assessment of public health capacity at the local level. Piloting the tool revealed some concerns amongst participants, particularly about how the tool would be used. However there was also recognition that the areas covered by the tool were those considered relevant.

## Background

Although public health in Australia involves numerous players, the major providers are governments. Australia does not have a dedicated ministry of public health or a national agency for public health, and at both a federal and state level responsibility for public health sits within a heath ministry. Australia has three levels of government - federal, state, and local, and each has some responsibility for planning and delivering public health services. The federal government provides funding, enacts legislation and sets policy direction [1]. States are responsible for the delivery of public health services and each jurisdiction is responsible for creating its own institutional arrangements for public health programs [2]. For a variety of historical and political reasons these organisational arrangements have evolved in distinct ways. Some states, such as Queensland have decentralised systems whilst others, such as Western Australia and South Australia have a more centralised systems [2]. The roles and responsibilities of local government are described in legislation set by state government and vary across jurisdictions.

Contemporary interest in public health systems and infrastructure is often associated with the release of the Institute of Medicine (IOM) report on the status of public health in the USA [3]. There are multiple definitions of infrastructure [4,5] and the National Public Health Partnership (NPHP) describe infrastructure as the "building blocks necessary to accomplish the activities of health protection, illness prevention and health promotion" [1].

If infrastructure constitutes the building blocks of the public health system, that system also requires capacity to perform its key functions. At the most basic level, capacity is simply the ability to produce or perform a product or service. However, there is no consensus about what, exactly, capacity means. Milen notes that there are now a wide range of both conceptual and operational definitions [6]. Beaglehole and Dal Poz describe public health capacity as "the ability to achieve stated public health objectives at the national, regional and global levels with respect to both ongoing and emerging health problems" [7] p.3). Milen provides the most comprehensive definition of capacity as "an ability to perform the defined functions effectively, efficiently and sustainably and so that the functions contribute to the mission, policies and strategic objectives of the team, organization and the health system" [6] p.4). In surveying a range of definitions, Jurie concludes that "capacity may be understood as the inherent endowment possessed by individuals or organizations to achieve their fullest potential. Capability would refer to the action taken on capacity in order to realize this potential" [8] p.271). What these definitions have in common is the concept of '*ability to*'.

Imbeau et al. argue that the sustainability of the health system requires financial, organizational and epistemic capacity [9]. Financial capacity is funding, both the total amount and the manner in which it is organized and delivered. They describe organizational capacity as "cohesive decision-making structures ... rules or standards", a concept that sounds rather like governance. Epistemic capacity refers to knowledge - both technical and normative [9] p.2-3). They argue that all three are essential and interdependent but that financial capacity tends to be privileged in discourses about system sustainability [9]. White, in a formulation that has some similarities, discusses political, technical and institutional capacity [10]. White's thesis is that the ability to lead change or address issues - in his case the USA federal government and the health system - relies on these three forms of capacity. Political capacity is the ability to garner political support, to build coalitions. Technical capacity refers to knowledge and the ability to identify an appropriate response, and institutional capacity is whether the government, or its agencies, have the power and resources to implement the change [10]. From these multiple definitions it appears that capacity is a collection of attributes and processes rather than a discrete entity and it implies a transformational ability.

Just as there is no agreement about what capacity is, nor is there agreement about exactly what capacity does. Milen argues that capacity is closely associated with performance, and poor performance may indicate capacity gaps[6]. Mittelmark et al. offer a slightly different formulation when they argue that "having the capacity to perform a task is an essential but not sufficient condition for good performance" [11]p.3). This suggests that the exact nature of the relationship between capacity and performance requires further investigation.

Discussions about capacity often also make reference to capacity building. Zonta and Wilson define the difference between the two as "capacityis a neutral word, conveying neither positive nor negative qualities. Capacity building, on the other hand, implies a deliberate effort to create, support or strengthen capacity" [12] p.27). Capacity building is a term that emerges in the development literature from an interest in reducing inequalities, maximizing donor aid and making the work of non-government organizations (NGOs) more effective. It has its history in concepts such as community development, participation and empowerment [13]. Use of the term is now common across a number of disciplines, but there is no agreed definition. In addition, Eade argues that capacity building is a term suffering from overuse and "is now used so indiscriminately that any meaning it once had may soon evaporate" [14] p.9). Capacity building is talked about in different ways - as a means to an end, as a process, or as an end in itself [14,15]. Hawe et al. argue that capacity building has three dimensions: building infrastructure; building partnerships and organizational environments to ensure sustainability; and building problem-solving capability [16] p.1). New South Wales Health's strategy for building capacity to improve health includes three areas for action: organizational development; workforce development; and resource allocation. They also identify leadership and partnerships as critical contextual issues [15].

### Efforts to map or measure capacity

Despite differences between capacity and capacity building, an assessment of existing capacity must be the first step in efforts to enhance capacity, particularly at the system level [17]. There have been efforts to develop tools to measure capacity within a variety of contexts - in different areas and at different levels. Capacity mapping may be concerned with specific components, for example workforce or leadership, or a particular health area. In other instances it may focus on specific levels, such as organizations, communities, the system or the national level. These different levels are related, although that relationship is difficult to define and measure.

Bush et al. have developed a tool to map community capacity [18]. Although this tool is not health specific, its focus on the local level and interest in how organizations may enhance the capacity of the community make it relevant to public health. They describe community capacity as "a collection of characteristics and resources which, when combined, improve the ability of the community to recognize, evaluate and address key problems" [18] p.1). Their interest is in identifying the "capacity available within a network of organizations and groups at the local level" [18] p.1). The Community Capacity Index (CCI) is built around four domains: network partnerships; knowledge transfer; problem solving; and infrastructure. The first three are measured by a set of indicators that result in an assessment of either first, second or third level capacity. The fourth, infrastructure, is measured by degrees of investment in policy, finance, human resources and social relations [18].

Catford suggests eight domains for measuring health promotion capacity at the national level: policies and plans; leadership; joined-up government; program delivery; partnerships; professional development; performance monitoring; and, sustainable financing. These might be measured on a five point scale that runs from "fully and effectively implemented" to "not currently actioned" [19] p.5). The World Health Organization (WHO), recognizing the importance of capacity mapping for health system strengthening, have funded efforts to map capacity for health promotion at the national level [20]. These tools are designed to capture information about the system's capacity to deliver, but they do not address community capacity.

In the USA, the most significant project has been the National Public Health Performance Standards Program (NPHPSP). Work began in 1997 by the CDCs Public Health Practice Program Office with a number of public health organisations as partners, to develop assessment instruments to measure public health system performance at both the state and local level. The project used the ten essential public health services (EPHS) as a framework because it was "developed through consensus ... [and was] a widely recognised and accepted model" [21] p.viii). A set of indicators was developed for each of the ten EPHS. They were finalised and released in 2002. A second version of the instruments has recently been released [22].

Prior to the release of the instruments, testing resulted in a number of recommendations, perhaps the most interesting being that "ongoing research is needed on the relationship of public health system capacity, performance and outcome" [23] p.196). Halverson argues that part of the value of the American performance standards lies in the fact that "what gets measured, gets done" [21] p.viii). It has been claimed that "the true value of the national performance standards rests with the ability of public health leaders to use these tools to strengthen system capacity" [24].

In 2002, the Centers for Disease Control and Prevention (CDC) developed a single-issue tool to assess emergency response capacity. It is built around six focus areas: planning and assessment; surveillance and epidemiology; laboratory capacity; information technology; risk communication; and education and training [25]. There are two versions, one for state level and the other for local health departments [25]. Local health departments collected baseline information in 2002 and it has been re-used in subsequent years to measure increases in capacity [26].

Dato et al. describe capacity mapping as "a strategy to find untapped and unrecognized resources" [27]. They suggest it as an approach for identifying public health training resources, given what they see as a mismatch between the need for public health training and the lack of funding [27]. Work by Hughes [28,29] on workforce capacity in public health nutrition and by Scanlon and Raphael [30] in mental health illustrate the interrelated nature of different areas and levels when attempting to map capacity. For example, Scanlon and Raphael argue that improving mental health will require increased capacity in three areas; the policy context, the workforce and the community [30].

Table 1 demonstrates some of the similarities and differences between domains/elements of public health and health promotion capacity. You would expect some differences because they relate to different levels. The PAHO [31] and Catford [19] works focus on the national level, the CDC [25] at the state or local level, whilst the Swiss work [32] is particularly concerned with capturing the context within which programs are implemented. There are however, commonalities with a shared concern for workforce issues, information systems and financing.

**Table 1:** Components of Capacity

PAHO Public Health National level	CDC Public Health Preparedness State and local level	HP Switzerland Health Promotion Program implementation level	J. Catford Health Promotion National level
Workforce	Education and training	Professionalization	Professional development

Information systems	Surveillance	Information systems	

Financial Resources		Resources	Sustainable financing

Organizational capacity	Laboratory capacity	Organizational capacity	

Technologies	Information technology		

	Planning and assessment	Policies, priorities and programs	Policies and plans
			
			Program delivery
			
			Performance monitoring
	
		Partnerships	Partnerships
		
		Leadership	Leadership
	
	Risk communication	Research	Joined-up government

Although there are important exceptions such as emergency preparedness, it is in health promotion, rather than public health more broadly, that the majority of the development work has been done (see for example [33,34,16,11]). The existing capacity assessment tools, discussed above, did not fit the requirements of our project. What we needed was an assessment tool that local agencies could use to provide a snapshot of the entire public health system. Some, such as the workforce framework developed by Dal Poz and others [35] or the work by Scanlon and Raphael [30], deal with only one issue or component. We felt that attempting to blend a number of different tools or frameworks would produce an unwieldy instrument that contained too great a degree of detail. The NPHPSP local instrument covers the field and it may be suggested that instrument was a logical choice. However, there are several reasons that this choice was rejected. The context is different and public health is not thought about or practiced in Australia in an identical way to that of the USA. Australia does not have a nationally agreed list that would equate with the 10 EPHS. In addition, the NPHPSP tool takes approximately 16 hours to complete [22] and it was felt that this "reporting burden" required a level of detail that went beyond that of a rapid assessment tool that, in the Australian environment, local public health people could reasonably be called upon to complete. Finally, and perhaps most importantly, the NPHPSP is a self named 'performance standards' instrument. Although the broad goals of systems improvement might be similar they represent different stages. Assessing capacity, as LaFond et al [17] noted, is a first step in efforts to improve capacity.

We felt it was important to develop an understanding of public health capacity at the local level. This is particularly vital in the Australian context for a number of reasons: the system is complex and there are significant differences in organizational arrangement across jurisdictions; funding for public health has generally remained static; and, a significant amount of public health is delivered at the local level. Developing a tool to assess the current capacity in the system is a first step in increasing our understanding of local public health capacity and the role it plays in the effective delivery of public health services.

## Methods

Australia has eight states and territories. Two states, New South Wales (NSW) and Victoria, were chosen. In population terms, these are Australia's largest states and so are responsible for delivering a significant proportion of Australia's public health services. In addition, as was noted earlier, the development of public health across Australian states has varied. As a result the infrastructure and capacity for public health is not uniform across jurisdictions. NSW and Victoria are illustrative of these differences in organisational arrangements. NSW has a centrally coordinated system, led by the state health department. Primary responsibility for the delivery of public health services rests with the Area Health Services (AHSs), which are state government agencies. Local government in NSW has a very limited role with respect to public health. In contrast, Victoria's system is relatively decentralised and fragmented. The state department of human services provides some direct services along with policy direction and funding for local service delivery, while a tobacco tax funded health promotion foundation also provides funding and technical guidance to local service providers. The 79 local governments play a key role in delivering public health services. Local governments have some degree of autonomy. However, they are created and governed by legislation at the state level. They have the authority to raise their own revenue but also rely on significant levels of funding from state government. At the same time, there are also local community health services, governed by independent boards of management, who have a role in aspects of public health, particularly in health promotion. For the purposes of this research the focus is on organisations who deliver the majority of public health at the local level, within their jurisdiction and who have a clear responsibility for doing so.

Once the organisations had been selected, the issue was determining who, within that organisation, should be spoken with. Within NSW, participants were Directors of Population Health, and the Managers of the Public Health and Health Promotion units. In Victorian local governments, appropriate participants were harder to identify because of the variety of ways in which these organisations are structured. The people selected were those in the most senior position with direct responsibility for public health. Most commonly these people were titled Community Health or Health Services Managers. In-depth interviews were conducted with over 20 senior managers. Following standard practices in qualitative research, sample size was not pre-determined, but rather interviewing stopped when saturation was reached, that is when the interviews stopped revealing new information.

Participants were asked to reflect on the critical components of infrastructure and capacity required at the local level. Each interview transcript was reviewed a number of times to identify reoccurring themes and sub-themes, in accordance with grounded theory methodology. This iterative process is necessary to ensure that themes, particularly those that do not appear in the earlier interviews, are not overlooked.

The information generated by the interviews was organized into broad categories which formed the basis of the capacity tool. Small workshops with public health experts in both states explored this data further. These people were selected on the basis of their recognized public health knowledge and experience, and their interest in systems improvement. They included both senior public health managers working in government as well as former senior managers. These workshops developed each category further and devised scores for each item. Both the interviews and the workshops also highlighted the need for two process issues to be considered. The first was that the tool must be based on self-assessment and its use be completely voluntary. The second was for it to be a rapid assessment that did not involve an onerous data collection requirement.

The instrument was piloted in meetings in Victoria and NSW. Participants were asked a variety of questions about the tool generally and about specific questions - for example: what was the degree of difficulty in completing the tool; were there critical areas that had not been included; and, would the tool, once finalized and disseminated, be useful for organizations?

The project had approval from La Trobe University's Human Ethic Committee (reference number 02-70). All participants indicated their willingness to take part, both verbally and in consent forms signed before the commencement of interviews.

## Results

The tool contains four categories. The first, *Policy Environment*, asks respondents about the broader system (see Table 2). This recognizes that the capacity of organizations working at the local level may be enhanced or constrained by the broader environment within which they operate. This category contains four elements: planning and strategic development; public policy; knowledge management; and leadership. Each of these elements asks respondents to consider a range of items. For example - within the leadership category the statement 'there are clearly identifiable leaders for public health' is posed. Respondents are asked to assess each item on a Likert-type response format, in the case of the example 'on all issues, on some issues, on few issues'. This means that scoring is possible. However, it is the assessment process itself that is important, particularly as a participatory exercise. The tool does not emphasize scoring or arriving at a numerical result because this should not be seen as the end objective.

**Table 2:** The Policy Environment

PLANNING AND STRATEGIC DEVELOPMENT AT THE SYSTEM LEVEL
Partnerships with other sectors

• Partnerships exist with other sectors whose work is relevant to, or impacts on, public health (*most relevant sectors---some relevant sectors ---few relevant sectors*)
• Partner organizations work together in a collaborative manner (*collaboration---consultation---information exchange*)
• Partnerships are institutionally embedded (*always---sometimes---rarely*)
• Where it is appropriate, these partnerships are sustained over time (*always---sometimes---rarely*)

Involvement with NGOs

• Partnerships exist with NGOs whose work is relevant to, or impacts on, public health (*most relevant organisations---some relevant organisations---few relevant organisations*)
• Partner organizations work collaboratively on planning and program delivery (*collaboration---consultation---information exchange*)
• Partnerships are institutionally embedded (*always---sometimes---rarely*)
• Where it is appropriate, these partnerships are sustained over time (*always---sometimes---rarely*)

Involvement with the private sector

• Partnerships exist with private sector organizations whose work is relevant to, or impacts on, public health (*most relevant organisations---some relevant organisations---few relevant organisations*)
• Partner organizations work collaboratively on planning and program delivery (*collaboration---consultation---information exchange*)
• Partnerships are institutionally embedded (*always---sometimes---rarely*)
• Where it is appropriate, these partnerships are sustained over time (*always---sometimes---rarely*)

Planning and resource allocation

• There are links between planning activities and resource allocation (*systematic links---informal links---no links*)

**HEALTHY PUBLIC POLICY**

Health sector

• In the health sector, legislation reflects contemporary public health philosophy and practice (*always---sometimes---rarely*)
• In the health sector, a policy statement or commitment to public health exists (*fully developed---partially developed---not developed*)
• This statement reflects contemporary public health philosophy and practice (*fully reflects---partially reflects---does not reflect*)
• Funding, training and coordination mechanisms are in place to implement policy (*fully in place---partially in place---not in place*)

Other sectors

• Legislation in other sectors, relevant to public health, contributes to public health goals and objectives (*always---sometimes---rarely*)
• Policy statements in other sectors, relevant to public health, contribute to public health goals and objectives (*always---sometimes---rarely*)

**KNOWLEDGE MANAGEMENT AT THE SYSTEM LEVEL**

Data and research

• High quality data is available (*always---sometimes---rarely*)
• High quality data is used in policy and program development (*always---sometimes---rarely*)
• High quality data is systematically appraised and used (*always---sometimes---rarely*)

Evaluation

• Evaluation findings are applied in policy and program development (*always---sometimes---rarely*)

Intellectual capital

• Specialist expertise is available (*always---sometimes---rarely*)
• Access to specialized expertise is facilitated where required (*always---sometimes---rarely*)

**LEADERSHIP AT THE SYSTEM LEVEL**

Advocacy for public health

• There are clearly identifiable leaders for public health (*on all issues---on some issues---on few issues*)
• These leaders provide a credible voice for public health (*on all issues---on some issues---on few issues*)
• These leaders set the agenda on issues relating to public health (*consistent agenda setting---episodic agenda setting---reactive only*)

Technical leadership

• Those in leadership and management positions provide the necessary technical leadership (*in all areas---in some areas---in few areas*)

Political commitment

• There is a political commitment to issues relating to public health (*strong commitment---commitment on some issues---weak commitment*)

The tool then asks respondents to consider three categories that relate specifically to their organization. The *Resources *(see Table 3) category covers human resources, financing and information systems. The *Programs *(see Table 4) category covers health protection, health promotion, and prevention activities undertaken by the organization. The final category, *Organizational Environment *(see Table 5) asks respondents to consider five dimensions within their organization: culture; leadership and management; partnerships; planning; and knowledge management.

**Table 3:** Organizational Resources

HUMAN CAPITAL AT THE ORGANISATIONAL LEVEL
Skills and competencies

• The organization has a policy on the basic skills it requires in its workforce (*explicit policy---informal policy---no policy*)
• These competencies form part of the criteria on first employment (*always---sometimes---rarely*)
• The organization is able to recruit staff with the skills and expertise appropriate for the position (*always---sometimes---rarely*)
• The organization has a policy on staff development opportunities (*explicit policy---informal policy---no policy*)
• Staff development opportunities are planned and occur (*regularly---occasionally---rarely*)
• The organization has formal links with population health training organizations (for example universities) (*formal links---informal links---no links*)

Workforce composition

• The organization's workforce is representative of the catchment population (*fully representative---partially representative---not representative*)

**FINANCING AT THE ORGANISATIONAL LEVEL**

• There is stability in the organization's financing arrangements (*assured and ongoing---roll-over---unpredictable*)
• The budget timeframe enhances the organization's ability to undertake planning (*long-term planning/5-10 years---medium-term planning/2-3 years---limited ability to plan*)
• Budgetary planning and management processes are incorporated into organizational and program planning (*fully incorporated---partially incorporated---not incorporated*)
• The organization has the ability/opportunity to make autonomous decisions (*high autonomy---some autonomy---low autonomy*)

**INFORMATION SYSTEMS AT THE ORGANISATIONAL LEVEL**

Activities data

• Comprehensive data is gathered about the organization's own activities (*always---sometimes---rarely*)

Health surveillance

• Comprehensive health surveillance activities are undertaken (*always---sometimes---rarely*)

Community characteristics

• Comprehensive data about the characteristics/demographics of the catchment community is gathered (*always---sometimes---rarely*)

Integrated systems

• Systems for information management are in place (*integrated systems---partially developed systems---no systems*)
• Analysis of gathered data is undertaken (*regularly---occasionally---infrequently*)

**Table 4:** Organizational Programs

ORGANISATIONAL HEALTH PROTECTION ACTIVITIES
Regulatory function

• The organization responds to its regulatory activities functions (*proactive and systematic response---systematic response---ad hoc response*)

Outbreak investigation

• The organization provides a proactive and extensive response to outbreaks (*always---sometimes---rarely*)
• The organization is able to assist other organizations with outbreak investigations (*often---occasionally---never*)

Disaster management

• The organization provides a proactive and extensive response to emergencies (*always---sometimes---rarely*)
• The organization is able to assist other organizations with disaster management (*often---occasionally---never*)

Disease surveillance

• There is a system for the active identification of emerging issues (*fully developed---partially developed---no system*)

**ORGANISATIONAL HEALTH PROMOTION ACTIVITIES**

Social and environmental factors

• The organization addresses social and environmental influences on health (*multiple strategies at different levels---some planned activities---on an ad hoc basis*)

• Strategies achieve measurable improvements (*always---sometimes---rarely*)

Family and community influences

• The organization addresses family and community influences on health (*multiple strategies at different levels---some planned activities---on an ad hoc basis*)
• Strategies reach target populations (*always---sometimes---rarely*)
• Strategies achieve measurable improvements (*always---sometimes---rarely*)

Individual lifestyle issues

• The organization addresses individual lifestyle issues (*multiple strategies at different levels---some planned activities---on an ad hoc basis*)
• Strategies reach target populations (*always---sometimes---rarely*)
• Strategies achieve measurable improvements (*always---sometimes---rarely*)

Health educations (individual and/or group)

• The organization provides health education to individuals and/or groups (*systematic approach---relies on interest of individual clinicians---no health education*)

**ORGANISATIONAL PREVENTION ACTIVITIES/PREVENTATIVE HEALTH SERVICES**

Screening

• The organization provides a screening service (*proactive outreach---organised---opportunistic*)
• The service includes efforts to reach populations at greater risk or those with access problems (*comprehensive targeted efforts---some targeted efforts---no targeted efforts*)

Immunization

• The organization provides an immunization service (*proactive outreach---organised---opportunistic*)
• The service includes efforts to reach populations at greater risk or those with access problems (*comprehensive targeted efforts---some targeted efforts---no targeted efforts*)

Maternal and child health

• The organization provides maternal and child health services (*proactive outreach---organised---opportunistic*)
• The service includes efforts to reach populations at greater risk or those with access problems (*comprehensive targeted efforts---some targeted efforts---no targeted efforts*)

Chronic disease self management

• The organization provides a chronic disease self-management service (*proactive outreach---organised---opportunistic*)
• The service includes efforts to reach populations at greater risk or those with access problems (*comprehensive targeted efforts---some targeted efforts---no targeted efforts*)

Reproductive health/family planning/STI services

• The organization provides reproductive health services (*proactive outreach---organised---opportunistic*)
• The service includes efforts to reach populations at greater risk or those with access problems (*comprehensive targeted efforts---some targeted efforts---no targeted efforts*)

**Table 5:** Organizational Environment

ORGANISATIONAL CULTURE
•The organization maintains a balance between being forward looking and maintaining operational stability (*always---sometimes----rarely*)
• The organization maintains a balance between risk taking and risk aversion (*always---sometimes----rarely*)
• The organization maintains a balance between a population health/community focus and direct service delivery (*always---sometimes----rarely*)
• The organization values and utilizes intellectual resources and capacity (*always---sometimes----rarely*)
• There are mechanisms in place for encouraging and supporting staff (*formal mechanisms---informal mechanisms---no mechanisms*)
• These mechanisms are engaged (*fully engaged---partially engaged---not engaged*)
• The organization has a policy on respecting diversity (*explicit policy---informal policy---no policy*)
• This policy is demonstrated in the organization's operational activities (including recruitment and program development) (*always---sometimes----rarely*)

**ORGANISATIONAL LEADERSHIP AND MANAGEMENT**

Vision and strategic direction

• The organization's leadership/management provide visible and convincing strategic direction (*always---sometimes---rarely*)
• The organization's vision/mission/strategic direction is clear to all staff (*all staff---some staff---few staff*)
• The organization's vision/mission/strategic direction is communicated to the broader community/catchment population (*extensively communicated---partially communicated---not communicated*)

Inter-face management

• The organization's leadership/management demonstrate the ability to manage the multiple external pressures facing the organization (*always---sometimes---rarely*)
• The organization's leadership/management act as a buffer between staff and external pressures (*always---sometimes---rarely*)

Innovation

• Innovation is valued within the organization (*explicitly valued---encouraged---discouraged*)
• Innovation receives strategic support from leadership/management (*consistently---occasionally---rarely*)

Prevention (population thinking)

• Prevention/population thinking is valued within the organization (*explicitly valued---encouraged---discouraged*)
• Prevention/population thinking receives strategic support from leadership/management (*consistently---occasionally---rarely*)

**PARTNERSHIPS AT THE ORGANISATIONAL LEVEL**

Intra-organizational

• There are links between units/departments within the organization (*formal links---informal links---no links*)
• Units/departments within the organization work together in a collaborative manner (*collaboration---consultation---information exchange*)

Other organizations (public sector)

• The organization has links with relevant public sector organizations (*most organisations---some organisations---few organisations*)
• Partner organizations work collaboratively on planning and program delivery (*collaboration---consultation---information exchange*)
• Partnerships are institutionally embedded (*always---sometimes---rarely*)
• Where it is appropriate, these partnerships are sustained over time (*always---sometimes---rarely*)

Other organizations (community)

• The organization has links with relevant community and private sector organizations (*most organisations---some organisations---few organisations*)
• Partner organizations work collaboratively on planning and program delivery (*collaboration---consultation---information exchange*)
• Partnerships are institutionally embedded (*always---sometimes---rarely*)
• Where it is appropriate, these partnerships are sustained over time (*always---sometimes---rarely*)

Other organizations (private sector)

• The organization has links with relevant community and private sector organizations (*most organisations---some organisations---few organisations*)
• Partner organizations work collaboratively on planning and program delivery (*collaboration---consultation---information exchange*)
• Partnerships are institutionally embedded (*always---sometimes---rarely*)
• Where it is appropriate, these partnerships are sustained over time (*always---sometimes---rarely*)

**PLANNING AND MONITORING AT THE ORGANISATIONAL LEVEL**

Systems

• The organization has a systematic approach to planning (*comprehensive and explicit systems---partially developed systems---limited systems*)
• There are links between the organization's planning, service delivery and management activities (*explicit and systematic links---partially developed links---limited links*)

Data and evidence

• The organization is able to access the data required for policy and program development (*always---sometimes---rarely*)
• The organization has a policy on the value of using evidence (*explicit policy---informal policy---no policy*)
• This policy is demonstrated in the organizations activities (*always---sometimes---rarely*)

Environmental scanning

• Comprehensive environmental scanning activities are undertaken to identify emerging public health issues (*always---sometimes---rarely*)

Evaluation

• The organization undertakes evaluation activities (*comprehensive and systematic---most activities---on an ad hoc basis*)
• This includes learning from both success and failure (*always---sometimes---rarely*)

Stakeholder/community involvement

• The organization has a policy on stakeholder/community involvement (*explicit policy---informal policy---no policy*)
• This policy is demonstrated in the organization's activities (including program development) (*always---sometimes---rarely*)

**KNOWLEDGE MANAGEMENT AT THE ORGANISATIONAL LEVEL**

Dissemination/sharing of knowledge

• There are mechanisms in place to ensure the organization keeps abreast of current research (*formal institutional mechanisms---informal mechanisms---no mechanisms*)
• The mechanisms are engaged (*fully engaged---partially engaged---not engaged*)
• There are mechanisms in place to encourage and facilitate the sharing of knowledge (*formal institutional mechanisms---informal mechanisms---no mechanisms*)
• These mechanisms are engaged (*fully engaged---partially engaged---not engaged*)

Intellectual capital

• There are mechanisms in place to encourage and facilitate the preservation and use of intellectual capital within the organization (*formal institutional mechanisms---informal mechanisms---no mechanisms*)
• These mechanisms are engaged (*fully engaged---partially engaged---not engaged*)

There is a second, much smaller part of the tool that asks a set of questions that are designed to provide some additional information about the organization. This allows participants to record contextual information that may impact on the organization's capacity. It includes questions such as: the size of the organization's current workforce; if problems have been experienced filling vacancies; the organization's core, ongoing program areas; and, the size of their budget. This section was included because any interpretation, particular if comparisons were to be done across organizations, needed to take account of the organization's circumstances and constraints.

There was general agreement amongst those completing the pilot that the main areas covered by the tool are important and relevant to public health at the local level. No items were deleted from the tool on the grounds that they were not required. However, there were suggestions that two additional areas were required with respect to individual organizations. Participants suggested that the tool be amended to make a distinction between a partnerships with NGOs and partnerships with the private sector, because they believed that these were different categories. The tool now asks those completing it to comment on their organization's partnerships with other public sector organizations, with community organizations, and with private sector organizations. Participants also wanted to capture an organization's ability to access population health training from external providers, such as universities, was seen as important. The tool now asks, as part of the Human Capital section, whether an organization has formal links with population health training organizations. One of the issues raised in the piloting of the tool in Victoria was its ability to capture the work of intermediary organizations - which in the case of Victoria are Primary Care Partnerships (PCPs). This is likely to be more of a concern in areas such as Victoria where public health involves multiple players.

Participants in the pilot also express concerns about the context for the tool's use, if it were to be officially sanctioned in policy. These included: would agencies by compelled to use it? Would they be compared against each other? Would this be a form of performance assessment by government? Despite these concerns, participants also indicated they saw the value in a capacity assessment tool for purposes of strategic planning.

## Discussion

Capacity mapping represents an important step in strengthening systems and their ability to deliver on core functions. In addition, as Fawkes and Lin note, despite higher costs in terms of time and money, capacity mapping where it is based on a dialogue method of data collection provides a number of additional benefits [20]. These include, inter alia, shared understandings about concepts and increased commitment [20] p.21). These are benefits that have been noted elsewhere [36]. However, the concerns raised by the participants in this study highlight a number of important questions: about the viability of mapping capacity; about quality; and, about culture in public health.

Public health is a broad and complex field, and developing a tool to assess system capacity is challenging though potentially rewarding. There are some questions relating to the validity of the tool, particularly as it attempts to operationalize a number of intangible concepts such as organizational culture. In the first instance, however, the key questions for tool development are: do the categories within the tool cover those factors critical to local public health?; do the questions within each category capture the important dimensions of the concept?; and, do the scales provide meaningful answers to those questions? These questions cannot be answered conclusively because the tool has only been piloted. More evidence will be accumulated as the tool is used in other areas, and this will help make an assessment. However, there are reasons to be confident that the tool does measure public health capacity. It is consistent with the literature on both infrastructure and capacity. In addition, feedback from the piloting suggested that the tool did capture the essential elements of capacity in a language and approach that made sense to the participants, despite differences in the framework for health administration.

Developing and implementing a systems tool in an environment where complex organizations are delivering complex services and where there are significant organizational and cultural differences between jurisdictions also presents challenges. Attempts to deal with these issues vary. The CDC capacity tool allows for some flexibility in what is measured, for example states may add location-specific questions to the capacity inventory [37]. Other tools employ flexible devices such as the spidergram [16,38] or the spokes and wheel diagram [19] in an attempt to capture and compare the multiple features. In the case of this tool, the issue can be addressed where organizations within a defined geographical location work together, after each completes the tool independently, to collate their information and so build a composite picture of public health capacity within their area.

Capacity assessment may be more useful as a baseline for capacity building, than for performance measurement. As such, it may be a useful part of a quality improvement system. In discussing a quality framework for public health in Australia, Swerissen outlines four possible processes: Continuous Quality Improvement/Total Quality Management (CQI/TQM); quality assurance; benchmarking; and performance measurement and monitoring [39]. There is an extensive literature on quality, and CQI and TQM are not generally seen as synonymous. Although mapping capacity may be designed to identify areas of strength and weakness and/or to discover existing resources, all with the broad aim of making improvements, it does not fit neatly into any of Swerissen's four categories. It is not quality assurance per se, nor is it performance measurement. Swerissen describes CQI/TQM as an ongoing process "that everyone in the organization is involved and concerned with improving the quality of an organization's products or service" whilst benchmarking is "the continual comparison and measurement of one organization's services and practices with others that undertake similar operations, but who are known in their field for excellence" [39] p.6-8). This tool is designed as a self-assessment instrument to enable organizations working in public health to map the capacity available to them. Therefore, it is not CQI/TQM or benchmarking - indeed, local public health managers expressed concerned about its potential use by government for benchmarking and performance measurement. Ideally, groups of organizations would gather baseline data and re-use the tool at a later date to identify changes in capacity over time. In that sense, using this tool could form a part of an organization's efforts to improve quality.

Although participants could see the value in mapping capacity, a number felt it was unlikely to be utilized unless there was a lead agency, or organizations were compelled to complete it. Whilst the notion of a lead or champion agency is a positive one, compulsion by government is unlikely to be a successful strategy. If capacity mapping is an important first step in strengthening the public health system by identifying priorities for building capacity, then its value rests with local organizations understanding the context for its adoption and application.

A more effective approach to implementation of the tool lies in recognizing and attempting to overcome the barriers to uptake. The primary barrier would appear to be the cost to organizations in terms of time. This would be exacerbated where organizations are funded to deliver public health programs but not for less tangible, but equally important areas, such as infrastructure and capacity. As Evans argues, with reference to public health in the UK, government rhetoric about the importance of capacity building has not been matched by funding. In addition he notes, efforts have tended to focus on public health specialists at the expense of those individuals and organizations who make a significant contribution to public health although it is not their primary business [40].

Another concern raised by participants was that the tool, although it was designed to map capacity, could be used as a form of performance measurement. This issue is one also identified by Ebbesen et al. in mapping health promotion capacity in Canada. They suggest that it may be addressed by researchers working to develop trust with those involved [41] p.89). The issues with this capacity tool are different given that it will be self-administered, which means participants should have greater control over the collection and use of the data. That this instrument is a self-assessment tool is an important and deliberate feature. Self-assessment may be criticised because it relies on the accurate reporting of those involved. This tool is designed for local organisations to assess their public health capacity and identify areas of strength and priority areas for capacity development. It is not a performance assessment tool. Participants would have nothing to gain by reporting in anything other than an accurate manner.

Some participants believed the tool might have most value as an aid to strategic planning. Several participants, in both states, suggested that the tool might be most useful in rural areas where resources and capacity are the most constrained. One participant suggested that metropolitan areas have a degree of "latent capacity". This is a concept found most often in business literature where it may refer to either unrecognized capacity that could be harnessed, or a form of surge capacity. Health Canada describe surge capacity as the resources and ability to continue to conduct business as usual and, at the same time, respond to an emergency [42].

The slow adoption of CQI in public health, relative to other areas of health, may suggest a culture that is not particularly reflective. In discussing obstacles to improving public health, Coye identified a range of issues [43]. She argued that internal factors included a resistance to accountability, a lack of understanding about relevant systems and a culture that emphasized command and control over influence and leadership. Public health would, she argued, be more successful if it adopted a systems thinking approach and developed a culture of continuous improvement [43]. Some of the responses from those piloting this tool, such as a suspicion about its purpose and the suggestion that organizations be compelled to complete it, illustrate Coye's points. However, it would be wrong to suggest that the concerns raised by participants simply reflect some form of cultural deficit. The public sector in Australia has been subject to successive waves of reform and organizational change. Public health organizations have faced budget cuts and job losses. In such an environment it is not surprising that people are wary.

## Next Steps

The most significant challenge to this tool will be encouraging organization to use it. All the people who participated in this project agree that well developed infrastructure and capacity are essential to the success of their efforts. The majority would also agree that assessing capacity, to identify strengths and weaknesses, is valuable. However, impediments exist. One is, as discussed earlier, a concern that it is a clandestine form of performance measurement, and this might be a particular problem given the power differential between organizations at the local level and state or federal organizations. A second barrier is the time involved in completing the tool. Although this instrument has been designed as a rapid assessment it will still require an investment, and organizations at the local level are already subject to many administrative demands.

## Conclusion

Despite the absence of a consensus about the exact definition of capacity, most authors agree that it involves the ability to take the action required to meet key functions. Most also agree that mapping current capacity is an important part of any attempt to improve it. The piloting of this tool highlighted some issues. There was wariness amongst some participants who were concerned that it was a form of performance measurement. However, an instrument that allows organizations to assess their public health capacity provides an opportunity for them to identify strengths and areas for improvement. These benefits are enhanced when the tool is completed by a process of dialogue within and between organizations. The components of the tool reflect those elements identified as important by those working in the field - a feature recognized and appreciated by those completing it. This discussion, about the process of developing and refining the tool, and its strengths and weaknesses, is another step in its dissemination. It is important to get this work right. A well functioning public health system requires infrastructure and capacity, but they are areas where it is difficult to attract and maintain funding. In part, this is because the level of capacity required across different components to ensure high performance is not known. Ultimately, its success will rely on its perceived usefulness and on the willingness of organizations to invest the time required to apply it. Organizational champions would make a significant difference to its uptake. It would sit best in a suite of continuous improvement activities, where it was repeated at appropriate intervals. There is no question that attempting to map public health capacity is a complex activity. However, it does represent a vital step in efforts to enhance or build capacity within the public health system at the local level.

This tool is comprehensive but imperfect. Even if it were possible to develop a tool that would capture every nuance of capacity, in every situation, there needs to be a balance between what is feasible in terms of resource investment, and gathering information that is robust and useful. Significantly, in their three state comparison, Pezzino et al. note that "an important finding from this project was that even an imperfect tool like the capacity inventory ... can produce valuable results." [26] p.vi).

## Competing interests

The authors declare that they have no competing interests.

## Authors' contributions

PB conducted the literature review, undertook data collection and analysis, contributed to the development of the tool and drafted the paper. VL conceptualised the study, contributed to the development of the tool, provided framing for the paper and commented on drafts.

## Pre-publication history

The pre-publication history for this paper can be accessed here:

http://www.biomedcentral.com/1471-2458/9/413/prepub
